# The effects of urban green space and road proximity to indoor traffic-related PM_2.5_, NO_2_, and BC exposure in inner-city schools

**DOI:** 10.1038/s41370-024-00669-8

**Published:** 2024-04-13

**Authors:** V. N. Matthaios, I. Holland, C. M. Kang, J. E. Hart, M. Hauptman, J. M. Wolfson, J. M. Gaffin, W. Phipatanakul, D. R. Gold, P. Koutrakis

**Affiliations:** 1 Department of Environmental Health, Harvard T.H. Chan School of Public Health, Boston, MA USA; 2 Department of Public Health Policy and Systems, University of Liverpool, Liverpool, UK; 3 Channing Division of Network Medicine, Department of Medicine, Brigham and Women’s Hospital, 181 Longwood Avenue, Boston, MA USA; 4 Harvard Medical School, Boston, MA USA; 5 Division of General Pediatrics, Boston Children’s Hospital, Boston, MA USA; 6 Division of Pulmonary Medicine, Boston Children’s Hospital, Boston, MA USA; 7 Division of Immunology, Boston Children’s Hospital, Boston, MA USA

**Keywords:** Air pollution, Child exposure/Health, Particulate matter, Environmental monitoring

## Abstract

**Background:**

Since there are known adverse health impacts of traffic-related air pollution, while at the same time there are potential health benefits from greenness, it is important to examine more closely the impacts of these factors on indoor air quality in urban schools.

**Objective:**

This study investigates the association of road proximity and urban greenness to indoor traffic-related fine particulate matter (PM_2.5_), nitrogen dioxide (NO_2_), and black carbon (BC) in inner-city schools.

**Methods:**

PM_2.5_, NO_2_, and BC were measured indoors at 74 schools and outdoors at a central urban over a 10-year period. Seasonal urban greenness was estimated using the Normalized Difference Vegetation Index (NDVI) with 270 and 1230 m buffers. The associations between indoor traffic-related air pollution and road proximity and greenness were investigated with mixed-effects models.

**Results:**

The analysis showed linear decays of indoor traffic-related PM_2.5_, NO_2_, and BC by 60%, 35%, and 22%, respectively for schools located at a greater distance from major roads. The results further showed that surrounding school greenness at 270 m buffer was significantly associated (*p* < 0.05) with lower indoor traffic-related PM_2.5_: −0.068 (95% CI: −0.124, −0.013), NO_2_: −0.139 (95% CI: −0.185, −0.092), and BC: −0.060 (95% CI: −0.115, −0.005). These associations were stronger for surrounding greenness at a greater distance from the schools (buffer 1230 m) PM_2.5_: −0.101 (95% CI: −0.156, −0.046) NO_2_: −0.122 (95% CI: −0.169, −0.075) BC: −0.080 (95% CI: −0.136, −0.026). These inverse associations were stronger after fully adjusting for regional pollution and meteorological conditions.

**Impact statement:**

More than 90% of children under the age of 15 worldwide are exposed to elevated air pollution levels exceeding the WHO’s guidelines. The study investigates the impact that urban infrastructure and greenness, in particular green areas and road proximity, have on indoor exposures to traffic-related PM_2.5_, NO_2_, and BC in inner-city schools. By examining a 10-year period the study provides insights for air quality management, into how road proximity and greenness at different buffers from the school locations can affect indoor exposure.

## Introduction

Primary schools are the second most important indoor micro-environment for children (other than homes) representing a unique and important location. Exposures to traffic-related pollutants in school may have a substantial impact on health of children who typically are in classrooms for over 6 h/day [1]. Schools that are located in close proximity to busy roads have higher exposures to traffic-related air pollutants (TRAPs) due to daytime traffic peaks and during morning and afternoon drop off/pick up times [2, 3]. Exposure to TRAPs at school has been associated with a range of adverse health impacts including respiratory conditions [4–6] and impaired neurodevelopment in schoolchildren, which contribute to a considerable personal and societal burden. In the USA, traffic-related sources account for 48% of the transportation PM_2.5_ and are the largest source of NO_*x*_ in an urban environment [7] accounting for 55%. There have been several studies that highlight the impact of road proximity to indoor traffic-related pollutants [8–10]. However, the quantitative aspect and the mitigation means of this association are less explored in school environments.

The implementation of green infrastructure is increasingly recognized as a promising strategy for mitigating the negative impacts of traffic pollution. There is mounting evidence that proximity of residence to city parks and regular visits to green spaces are associated with health benefits, including physical activity, social coherence, and stress reduction pathways [11, 12]. Greenness around schools is thought to improve health through direct effects on cognitive restoration and stress reduction [13] as well as by mitigating exposure to air pollution, noise, and extreme temperatures [14–16]. Past studies have shown that greenness provides the beneficial effects of lowering levels of both outdoor [17, 18] and indoor PM_2.5_ at residences [19] and schools [20]. However, these findings are inconsistent, and show strong regional heterogeneity [21, 22]. As a concept, green space represents diverse landscape features in myriad arrangements, with a variety of functionalities [13]. Consequently, the interaction between green infrastructure design (i.e., species selection, spatial positioning) and air pollutants can either positively or negatively affect personal exposure and thus human health [23].

Since traffic-related pollutants such as PM_2.5_, BC, and NO_2_ have been associated with adverse health effects, any amount of exposure reduction due to green space may provide benefits to children whose respiratory systems are still developing [14, 24]. In this study, conducted in a north-eastern USA city, we evaluated the long-term impacts of road proximity and quantified greenness levels in an inner-city environment, on the traffic-related air pollutant exposures inside school classrooms. This study is building upon the data from The School Inner City Asthma Study I & II (SICAS 1 & 2) where previously published results have identified and quantified the controllable factors and sources that influence outdoor and indoor air pollution exposures [2, 25]. How proximity to major roadways at home and school increase asthma symptoms [26] and how NO_2_ levels inside classrooms even at low levels affect airflow obstruction [6].

## Methods

### Study design

SICAS1 and SICAS2 investigated the effect of school- and classroom-based environmental exposures on students with asthma in a city in the north-eastern USA. The study spanned 10 years 2008–2014; 2015–2019 and included classroom measurements throughout the academic school. Weeklong indoor NO_2_, PM_2.5_, and BC measurements were conducted in inner-city school classrooms during weekdays, incorporating both occupied and non-occupied periods. SICAS1 is a prospective study evaluating the school/classroom-specific risk factors and asthma morbidity among urban children. It included 350 elementary school-aged children with asthma from multiple classrooms in 38 inner-city schools. Recruitment was ongoing for 5 years and started in spring 2008 with a follow-up sampling in autumn or winter. SICAS2 was a randomized control trial of 247 students with asthma to test a school-classroom level intervention in 41 schools, where the baseline measurements occurred between October and November, the first follow-up December–February, and the second follow-up between March and May. In total, 309 unique classrooms of unique 74 schools were studied here. The rationale of the studies is described in detail elsewhere [27, 28].

### Air pollution measurements

Weekday-period indoor PM_2.5_ samples were collected on Teflon filters at a flow rate of 1.8 L/min [29]. A total of 518 indoor PM_2.5_ samples were collected during the study period. PM_2.5_ collected on Teflon filters was measured gravimetrically with an electronic microbalance (MT-5 Mettler Toledo, Columbus, OH) in a temperature/RH-controlled room, following USEPA guidelines. Indoor filters were also measured for BC concentrations using a Smokestain Reflectometer (Model EEL M43D, Diffusion Systems Ltd., UK). Indoor NO_2_ was collected using Ogawa passive samplers and analyzed using ion chromatography. Concurrent daily outdoor PM_2.5_, BC, and NO_2_ concentrations were also measured at a central monitoring site. PM_2.5_ samples were collected using the Harvard Impactor [30], BC concentrations were measured using a single (*λ* = 880 nm) channel Aethalometer (model AE-16, Magee Scientific, Berkeley, CA), and NO_2_ was measured with chemiluminescent monitors. Indoor and outdoor samples were compared by matching the weekly indoor samples to the corresponding average daily outdoor samples. The median distance between the central site and schools was 4974 m. The contribution of traffic to PM_2.5_ concentrations was estimated with receptor modeling as described elsewhere [25]. This is a PM_2.5_ source that is derived by the PM_2.5_ mass and elemental composition using the positive matrix factorization modeling technique developed by USEPA [31, 32]. After the source apportionment 420 out of the 518 PM_2.5_ samples were found to have contributions from traffic sources. This traffic-related PM_2.5_ component (referred to as T-PM_2.5_) was abundant in organic and elemental carbon from motor exhaust, and metals from brake and tire wear (Fe, Cu, Zn). For traffic-related NO_2_ and BC we excluded samples when the indoor:outdoor ratio of NO_2_ was higher than 1.2 which is likely to indicate influence of indoor sources [33, 34].

### Urban green space

We estimated the greenness around schools by the use of Normalized Difference Vegetation index (NDVI). NDVI is a satellite-derived measure that captures photosynthetic activity of vegetation [35]. NDVI values range from −1.0 to 1.0, with negative values indicating clouds, snow and water, positive values near zero indicating bare soil, and higher positive values of NDVI ranging from sparse vegetation (0.1–0.5) to dense green vegetation (0.6 and above). NDVI data for every season between 2008 and 2019 at a 30 m resolution were obtained from Google Earth Engine [36]. Landsat 7 data were used for 2008–2013 and Landsat 8 for 2014–2019. We applied Google Earth Engine’s cloud cover algorithm to retain the least cloudy image within each season (January–March, April–June, July–September, October–December). NDVI within each season were assessed within 270 and 1230 m surrounding each geocoded school address, to represent viewable and walkable areas around each school. The 270 m buffer was selected to represent greenness directly accessible outside each school, while the 1230 m buffer represent a walkable distance buffer as reported in the literature [37].

### Distance to roadway

The distance between each school to the nearest primary or secondary road was calculated using Arc-GIS 10.2.2 (Environmental Systems Research Institute, Redlands, CA) software with 80% spelling sensitivity and 10-meter offset. First, a state-wide school database provided by the Office of Geographic Information to geocode school locations was mapped. We used a buffer of 300 m around each address to calculate the straight-line distance to the nearest primary and secondary roads [26]. Primary and secondary roads were defined using TIGER/Line files within the federal highway system. According to the US Census Bureau, primary roads are limited-access highways within the Federal interstate highway system or under state management with interchanges and accessible by ramps, including some toll highways. Secondary roads are main arteries, usually in the U.S. highway, state highway, or county highway system, with one or more lanes of traffic in each direction, may or may not be divided, and usually have at-grade intersections with many other roads and driveways.

### Statistical analysis

Following the approach of Dadvand et al. [21], we developed mixed-effects models for each pollutant, with schools included as a random effect to account for the repeated measurements within each school. We used weeklong indoor levels of each pollutant as the dependant variable and greenness in the 270 or 1230 m surrounding each school as a fixed-effect predictor. Given that schools were monitored in different weeks during each campaign period, we adjusted the analyses of each TRAP for the weekly average level of that TRAP (during the corresponding sampling week for each weeklong school indoor concentration with its corresponding from the central urban background site) measured by an urban background monitoring station to remove temporal fluctuation in background TRAP levels from our analyses [38]. Because we only had one background site when adjusting for background levels we included a random slope which represents the spatial relationship of each school to that site [2]. We further adjusted the models of indoor T-PM_2.5_, BC, and NO_2_ for outdoor concentrations, seasonality (cos*d* = cos (2 × *p* × *d*/365)) due to variations in greenness and differences in emissions, meteorological parameters (ambient temperature, wind speed, and boundary layer height), number of windows, and window orientation (facing toward bus drop off/pick up area), and school characteristics including building age and ventilation as fixed-effect predictors. The model is illustrated in the following equation:

$${Y}_{{ij}}={\beta }_{0}+{X}_{{ij}}{\beta }^{{\prime} }+{\mu }_{i}+{u}_{{ij}}+{\varepsilon }_{{ij}}$$

where ${Y}_{{ij}}$ represents the response variable weeklong indoor concentrations in school *i* and week *j*. ${X}_{{ij}}$ is a matrix of fixed effects predictors in school *i* and week *j* (i.e., outdoor concentration, boundary layer height, greenness, seasonality, etc.). ${\beta }^{{\prime} }$ is a vector of fixed-effect coefficient. ${\mu }_{i}$ is the random intercept representing school-specific variability (between classrooms). ${u}_{{ij}}$ is the random slope representing slope of the fixed-effect predictors that varies randomly across different schools. ${\varepsilon }_{{ij}}$ is the error term. The inclusion of both school-specific variations (${\mu }_{i}$) and variations in the slopes of the fixed effects across schools (${u}_{{ij}}$) can be particularly useful when the relationship between predictors and the response varies across different schools.

## Results

**Fig. 1:**
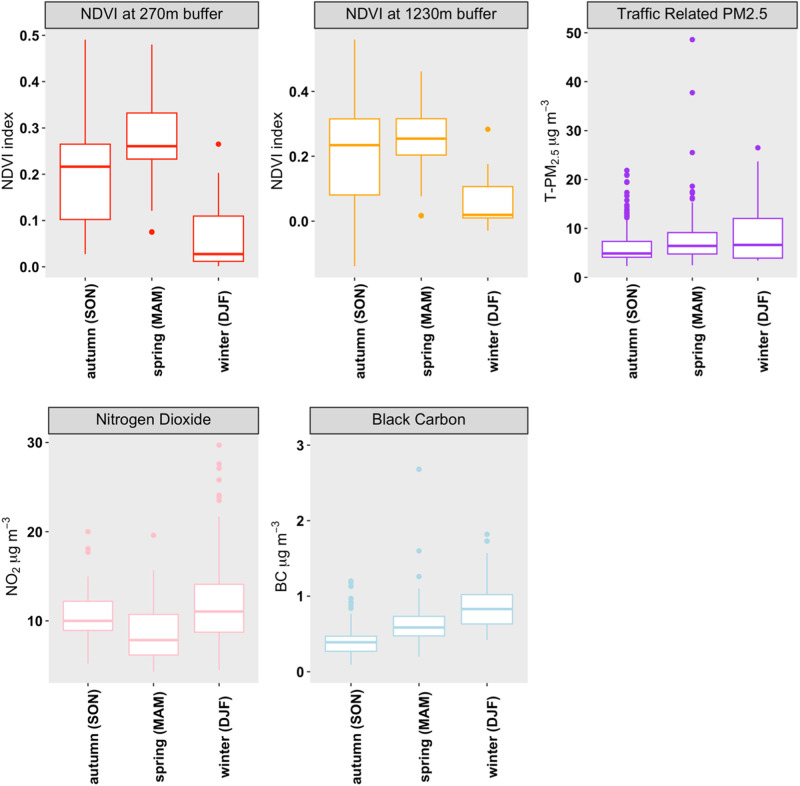
Seasonal levels of NDVI around schools and indoor T-PM_2.5_ (*n* = 420), NO_2_ (*n* = 351), and BC (*n* = 372) levels between 2008 and 2015; 2015–2019. Box parameters are the interquartile range (IQR), the hash mark is the median, and whiskers extend to 1.5 times the IQR above the 75th and below the 25th percentiles.

The median greenness around schools at 270 m between 2010 and 2019 was 0.204 (IQR = 0.163), with a mean value of 0.198 (s.d. = 0.122). It had a clear seasonal pattern with higher values during spring and lower values during winter. The mean (±s.d.) values were 0.217 (±0.107), 0.060 (±0.064), and 0.272 (±0.100) for autumn, winter, and spring, respectively (Fig. 1). Similar seasonal variations were observed with the 1270 m buffer. Figure 1 shows the seasonal variations of greenness and indoor T-PM_2.5_, NO_2_, and BC. Mean T-PM_2.5_ levels were 6.4 (±3.9), 8.8 (±5.7), and 8.4 (±6.8) for autumn, winter, and spring, respectively. Mean indoor NO_2_ concentrations were 10.5 (±3.3), 11.9 (±4.5), and 9.0 (±3.6) while BC levels were 0.40 (±0.2), 0.9 (±0.3), and 0.7 (±0.8) for autumn, winter, and spring, respectively.

**Fig. 2:**
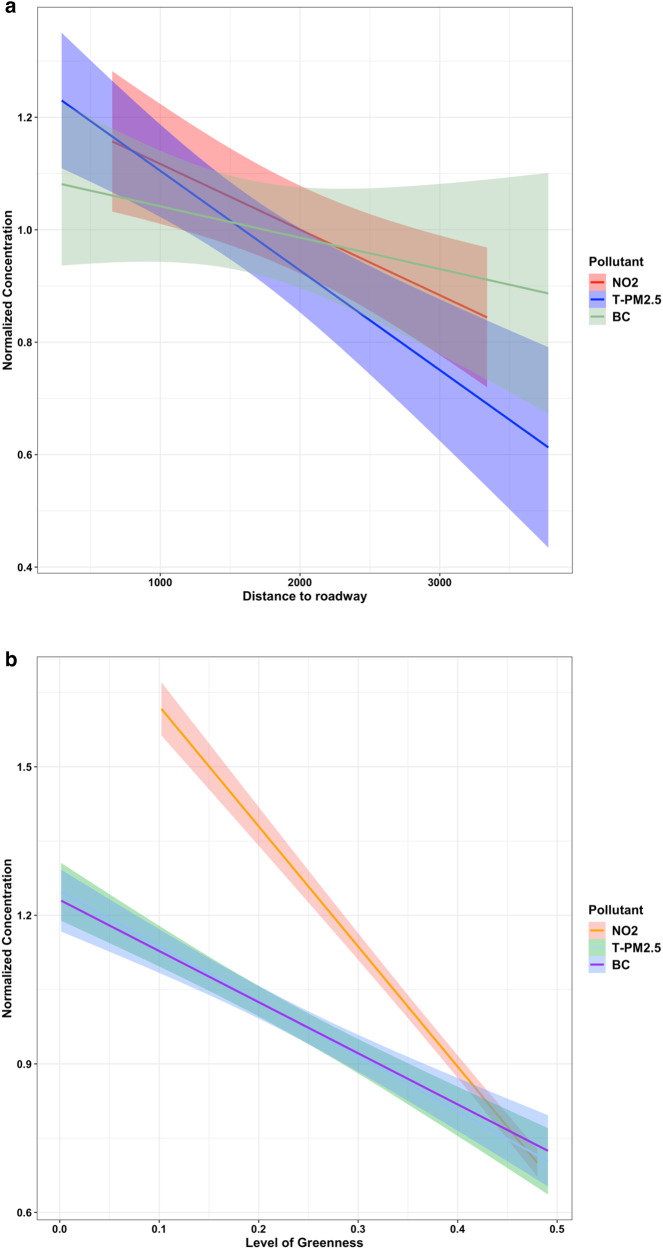
Effects of green space and road proximity. **a** Relationship between normalised indoor traffic-related air pollution and road distance, **b** relationship between normalised indoor traffic-related air pollution and greenness at 270 m. The shaded area indicates confidence intervals at 95%. T-PM_2.5_ (*n* = 420), NO_2_ (*n* = 351), BC (*n* = 372).

Figure 2 shows the relationship between the normalized (to the mean) indoor T-PM_2.5_, NO_2_, BC, and roadway proximity. It is evident that all traffic-related pollutants follow a linear decay with distance. The sharpest decrease with distance is for T-PM_2.5_ followed by NO_2_, while BC has a smoother decrease with distance. Schools that are more than 3 km away from roadways experience (on average) a 63%, 35%, and 22% decrease in T-PM_2.5_, NO_2_, and BC compared to those that are close to major roadways. Schools that have increased green areas directly accessible outside each school at 270 m showed great reductions in traffic-related pollutants by 64%, 61%, and 107% in T-PM_2.5_, BC, and NO_2_ compared to schools with little to no greenness. NO_2_ showed a sharper and more abrupt decrease with increased greenness while T-PM_2.5_ and BC showed similar less abrupt decreases. Similar decreases were found for the 1230 m buffer where NO_2_ decreased by 118% while T-PM_2.5_ and BC showed 68% and 67% reductions, respectively.

The results from the regression analysis of greenness and road proximity to traffic-related indoor PM_2.5_, NO_2_, and BC are shown in Table 1. For the road proximity in the unadjusted model there were inverse associations with T-PM_2.5_ and BC, however, they were not significant as in the case of NO_2_. After adjusting the models all relationships became significant (*p* < 0.05). Higher NDVI was statistically significantly (*p* < 0.05) associated with lower indoor T-PM_2.5_, NO_2_, and BC at a buffer of 270 m from schools. The coefficient of the association was stronger for greenness at a buffer of 1230 m from the schools. Analyses additionally adjusted for regional outdoor PM_2.5_, NO_2_, and BC, as well as meteorological parameters such as wind speed, temperature, boundary layer height, seasonality, and school characteristics: ventilation type and number of windows. The relationship between greenness and indoor T-PM_2.5_, NO_2_, and BC for the adjusted models had stronger coefficients for both 270 and 1230 m. Indoor T-PM_2.5_, NO_2_, and BC levels are influenced to some extent by regional pollution and the meteorological factors. Therefore, when controlling for these factors the associations between greenness and indoor T-PM_2.5_, NO_2_, and BC became stronger, allowing for a more accurate assessment of the effect of greenness and road proximity on indoor air pollution and minimizing the potential confounding effects of other variables.

**Table 1:** Regression coefficients and confidence intervals (CI) at 95% indicating the change of indoor T-PM_2.5_, NO_2_, and BC per NDVI increase at 270 and 1230 m of the school boundaries and road proximity.

Model	NDVI 270 m		NDVI 1230 m		Road distance	
	Coefficients (95% CI)	*p*	Coefficients (95% CI)	*p*	Coefficients (95% CI)	*p*
T-PM_2.5_						
Unadjusted	−0.068 (−0.124, −0.013)	<0.01	−0.101 (−0.156, −0.046)	<0.01	−5.48e-05 (−1.24e-04, −1.46e-05)	0.12
Adjusted^a^	−0.109 (−0.149, −0.068)	<0.01	−0.136 (−0.174, −0.098)	<0.01	−1.00e-04 (−1.48e-04, −5.39e-05)	<0.01
NO_2_						
Unadjusted	−0.139 (−0.185, −0.092)	<0.01	−0.122 (−0.169, −0.075)	<0.01	−1.21e-04 (−1.61e-04, −8.10e-05)	<0.01
Adjusted^a^	−0.550 (−0.843, −0.262)	<0.01	−0.361 (−0.656, −0.067)	0.016	−6.69e-05 (−1.03e-04, −3.07e-05)	<0.01
BC						
Unadjusted	−0.060 (−0.115, −0.005)	0.032	−0.080 (−0.136, −0.026)	0.004	−1.20e-05 (−8.01e-05, −5.64e-05)	0.18
Adjusted^a^	−0.069 (0.119, −0.019)	0.048	−0.103 (−0.147, −0.058)	<0.01	−8.62e-05 (−1.35e-04, −3.68e-05)	<0.01

^a^Adjusted model for regional pollution levels, wind speed, temperature, seasonality, number of windows, window orientation, and school ventilation.

## Discussion

The study investigated the associations of roadway proximity and greenness with exposures to traffic-related PM_2.5_, NO_2_, and BC inside inner-city schools. The analysis was based on 420, 362, and 372 PM_2.5_, NO_2_, and BC sample pairs, respectively that were collected indoors, in 74 inner-city schools, and outdoors, at a central urban background location. The results showed statistically significant declines of indoor traffic-related PM_2.5_, NO_2_, and BC with increases in roadway distance and greenness.

The decreases in T-PM_2.5_ exposure with road distance were greater than 60% for schools that are located 3 km away from roadways, while NO_2_ dropped by one third and BC by one fifth for the same schools. Amram et al. [39] showed similar findings for NO and ultrafine particles (both tracers for traffic-related pollution). These results show the importance of school location to indoor exposures to traffic-related air pollution which consequently have been shown to affect children’s lung development and health [40, 41]. Road proximity also affects indoor PM_2.5_ composition and elements related to tires, brakes, and road dust have more dramatic decreases [10]. Studies have also shown that road proximity of schools is associated with increased asthma symptom days for asthmatic children [26], while it has also been associated with increased childhood leukemia [42] and neurobehavioral disorders [43]. Furthermore, road proximity in general has been shown to influence birth outcomes [44] and has been associated with increased Parkinson’s disease risk [45], cognitive impairment [46], and with insidious effects on structural brain aging, even in dementia- and stroke-free persons [47].

The results further showed that surrounding school greenness was associated with a >60% reduction in traffic-related pollutants providing evidence for an urban level intervention to reduce children’s exposure to traffic-related air pollution. School greenness was also significantly associated with lower indoor traffic-related pollutants. These associations were stronger for surrounding greenness at a greater distance from schools highlighting the importance of open green areas at an inner-city environment. Open green areas can offer more protective means in buffering indoor school pollution levels by gradually reducing traffic-related pollution over space. Additionally, more green areas at a greater distance could also potentially mean fewer roadways. Our results were consistent with findings of previous studies reporting inverse associations between exposures to traffic-related pollutants inside schools and surrounding greenness at different distances from the school locations [21]. Improvements between indoor PM_2.5_ exposure in areas with high surrounding green space have been more consistent in the literature [48]. This link is straightforward since trees, shrubs, and hedges often act as filtration media (dry deposition) to outdoor PM_2.5_ [49, 50] and BC [51], however, their type (i.e., needle leaf, broad leaf, etc.), effective density, and porosity strongly influence their effectiveness [52, 53]. Even though there have been studies reporting NO_2_ reductions naturally onto vegetation [54, 55] or in artificial biofilters [56] via dry deposition, the reality is more complicated since more vegetation will result in greater biogenic volatile organic compound (BVOC) emissions in the near-school environment. These increased BVOC emissions are involved in complex photochemical reactions with NO_2_ and ozone (O_3_) and in the generation of secondary organic aerosols, therefore, might offset some of the health benefits of the overall greenness.

Previous studies that investigated factors that affect indoor school exposure to PM_2.5_, NO_2_, and BC showed that the biggest contributor to indoor air pollution is the infiltration of outdoor air pollution [2]. The association between surrounding greenness and indoor exposure to traffic-related pollution is, therefore, affected by the reductions of the outdoor traffic-related pollution with greenness. To examine that here, we fully adjusted our models for factors such as regional pollution and meteorological variables (such as wind speed, temperature), school ventilation practices, and seasonality. We found that the association between greenness and indoor exposure becomes stronger after adjusting for these variables, suggesting that part of the benefits inside schools are likely due to the benefits of the overall reduction of outdoor traffic-related pollutants by greenness. Surrounding school greenness, not only reduces overall traffic-related air pollution, but it can also improve cognitive performance of children [57], even when children are exposed only for short time periods to nature [58]. Other benefits related to residential greenness for children include lower obesity levels [20], fetal growth [59], reduction of the risk of cardiovascular disease [60], and decreased risk of cancer mortality [61].

Despite that our results can be considered robust, spanning 74 schools over 10 years, there are several limitations in this study. The data are from one region in north-eastern US and the findings might vary for other regions depending on the city/region – specific urban infrastructure, greens species variety (i.e., trees, shrubs, plants, etc.) and vehicle fleet emissions (i.e., proportion of heavy duty vehicles vs passenger cars vs light duty vehicles, diesel vs gasoline vehicles, etc.). In addition, the study did not include outdoor sampling directly outside the schools and instead included one regional background site location and surrogates to adjust for outdoor concentrations. To ensure consistency and robustness future studies should also consider expanding this approach by including multiple cities that can represent both north and south multinational environments as well as high-middle and low-middle income countries. Due to city-wide air pollution exposure disparities future studies should also consider if and how socioeconomic factors play a role in these associations.

## Implications for children’s exposure and recommendations for improvement

According to the World Health Organization (WHO), more than 90% of children under the age of 15 worldwide are exposed to air pollution levels exceeding the WHO’s recommended guidelines [62]. Given that children spend a significant portion of their day in school, it is crucial to enhance our knowledge regarding their exposure pathways to air pollution. In this large school indoor exposure study conducted in 74 schools across 10 years, the findings showed that children attending schools located near busy roads, not necessarily highways, and schools that have less nearby green infrastructure are exposed to greater traffic-related PM_2.5_, NO_2_, and BC and are likely at a greater health hazard risk. In a recent literature review regarding the air quality around schools, Osborne et al. [63] indicated that nearby traffic is a key determinant of concentrations outside schools and that factors related to planning and urban design such as green playgrounds, and amount of surrounding green space can reduce school site air pollution. Given the adverse health impacts of traffic-related air pollution and the improved health benefits of greenness, actions are needed to improve children’s health and well-being during their early years of development. Green infrastructure, natural or artificial via botanical biofilters, in and around schools as well as the creation of clean air zones at an effective road proximity from schools are two key reduction measures that local authorities, policy makers, school managers, and urban planners should evaluate and consider as unique or combined interventions in order to reduce children’s exposure to air pollution and improve their health and well-being.

## Data Availability

The data are available from the corresponding author on reasonable request.
